# Facilitators and Barriers to the Implementation of Digital Health Technologies in Hospital Settings in Lower- and Middle-Income Countries Since the Onset of the COVID-19 Pandemic: Scoping Review

**DOI:** 10.2196/63482

**Published:** 2025-03-06

**Authors:** Sheng Qian Yew, Daksha Trivedi, Nurul Iman Hafizah Adanan, Boon How Chew

**Affiliations:** 1 Department of Public Health Medicine, Faculty of Medicine National University of Malaysia Cheras Malaysia; 2 Centre for Research in Public Health and Community Care University of Hertfordshire Hertforshire United Kingdom; 3 Clinical Research Unit Hospital Sultan Abdul Aziz Shah Serdang Malaysia; 4 Faculty of Medicine and Health Sciences Department of Family Medicine Serdang Malaysia

**Keywords:** digital health implementation, facilitators, barriers, digital health classification framework, lower- and middle-income countries

## Abstract

**Background:**

Although the implementation process of digital health technologies (DHTs) has been extensively documented in high-income countries, the factors that facilitate and prevent their implementation in lower- and middle-income countries (LMICs) may differ for various reasons.

**Objective:**

To address this gap in research, this scoping review aims to determine the facilitators and barriers to implementing DHTs in LMIC hospital settings following the onset of the COVID-19 pandemic. Additionally, the review outlined the types of DHTs that have been implemented in LMICs’ hospitals during this pandemic and finally developed a classification framework to categorize the landscape of DHTs.

**Methods:**

Systematic searches were conducted on PubMed, Scopus, Web of Science, and Google Scholar for studies published from March 2020 to December 2023. We extracted data on authors, publication years, study objectives, study countries, disease conditions, types of DHTs, fields of clinical medicine where the DHTs are applied, study designs, sample sizes, characteristics of the study population, study location, and data collection methods of the included studies. Both quantitative and qualitative data were utilized to conduct a thematic analysis, using a deductive method based on the Practical, Robust Implementation and Sustainability Model (PRISM), to identify facilitators and barriers to DHT implementation. Finally, all accessible DHTs were identified and organized to create a novel classification framework.

**Results:**

Twelve studies were included from 292 retrieved articles. Telemedicine (n=5) was the most commonly used DHT in LMICs’ hospitals, followed by hospital information systems (n=4), electronic medical records (n=2), and mobile health (n=1). These 4 DHTs, among the other existing DHTs, allowed us to develop a novel classification framework for DHTs. The included studies used qualitative methods (n=4), which included interviews and focus groups, quantitative methods (n=5), or a combination of both (n=2). Among the 64 facilitators of DHT implementation, the availability of continuous on-the-job training (n=3), the ability of DHTs to prevent cross-infection (n=2), and positive previous experiences using DHTs (n=2) were the top 3 reported facilitators. However, of the 44 barriers to DHT implementation, patients with poor digital literacy and skills in DHTs (n=3), inadequate awareness regarding DHTs among health care professionals and stakeholders (n=2), and concerns regarding the accuracy of disease diagnosis and treatment through DHTs (n=2) were commonly reported.

**Conclusions:**

In the postpandemic era, telemedicine, along with other DHTs, has seen increased implementation in hospitals within LMICs. All facilitators and barriers can be categorized into 6 themes, namely, (1) Aspects of the Health Care System; (2) Perspectives of Patients; (3) External Environment; (4) Implementation of Sustainable Infrastructure; (5) Characteristics of Health Care Organization; and (6) Characteristics of Patients.

## Introduction

Populations residing in lower- and middle-income countries (LMICs) face numerous unmet health care needs due to various factors [1], including an aging population [2], escalating health care costs [3], widening income disparities [4], increased child morbidity and mortality [5], the emergence of new epidemics and pandemics [6], and growing racial discrimination in health care access [7]. The COVID-19 pandemic has further exacerbated the existing disparities and limitations of health care systems in LMICs, further exposing the issues of understaffing, underfunding, inadequate infrastructure, limited access to testing and treatment, and vulnerability to health emergencies [8]. In response to this unprecedented pandemic, many hospitals in LMICs have attempted to leverage digital health technologies (DHTs) as an innovative approach to curb the spread of the SARS-CoV-2, improve health care provision, and strengthen pandemic response efforts [9].

Generally, DHTs are defined as a set of information and communications technologies utilized in medicine and health care to manage illnesses and promote wellness [10]. These technologies have expanded as a transformative force in health care since the onset of the COVID-19 pandemic era, thereby offering a myriad of benefits that revolutionize health care delivery and improve patient outcomes. These technologies encompass a wide range of digital tools, including mobile health (mHealth) [11], telemedicine [12], wearable technologies [13], electronic medical records [14], big data analytics [15], Internet of Medical Things [16], blockchain in health care [17], metaverse [18], software as a medical device [19], augmented reality [20], and virtual reality [21]. With increasing computing power and appreciation of artificial intelligence [22] and machine learning [23] in health and medicine, many such smart tools are an making appearance in different aspects of hospital care [24,25].

Despite the benefits of DHTs, such as improved access to health care, enhanced patient engagement and empowerment, efficient health care delivery, timely and personalized care, remote monitoring, and data-driven decision-making, the implementation process of DHTs in the hospital settings, including its facilitators and barriers, is mostly described in the context of high-income countries [26,27]. Given the differences in resources, infrastructure, health care systems, socioeconomic status, level of digital divide, and regulatory frameworks in the LMICs compared with the high-income countries, the evidence on facilitators and barriers to implementing DHTs reported in previous literature may not apply to the LMICs [28]. To address these research gaps, this scoping review aimed to:

- Provide an overview of the facilitators and barriers in implementing DHTs within hospital settings in LMICs since the onset of the COVID-19 pandemic.
- Identify and describe the types of DHTs that have been put into practice in hospitals within LMICs since the onset of the COVID-19 pandemic.
- Develop a classification framework to better define the landscape of DHTs, providing a more comprehensive and practical understanding.

## Methods

### Design

The scoping review was conducted using the methodological framework developed by Arksey and O’Malley [29]. The protocol has been registered in the Open Science Framework and has been previously published [30]. The PRISMA-ScR (Preferred Reporting Items for Systematic Reviews and Meta-Analyses Extension for Scoping Reviews) [27,31] was used to conduct and report our findings.

### Identifying Relevant Literature

To comprehensively identify the literature relevant to DHTs, a broad, sensitive, and specific search strategy was applied to capture all DHT-related literature. With the assistance of an information technologist, a comprehensive list of literature relevant to DHTs was identified according to the criteria below. Textboxes 1 and 2 describe the inclusion and exclusion criteria adopted for this scoping review. The focus was on the period between March 2020 and December 2023, as the World Health Organization (WHO) officially declared COVID-19 as a pandemic in March 2020.

**Inclusion criteria.**

- Studies that were conducted in lower- and middle-income countries (LMICs). (The World Bank classifies countries by income each year, covering all nations with a population over 30,000. In 2023, countries are divided into 4 income categories based on their gross national income (GNI) per capita. Low-income countries are defined as those with a GNI per capita of US $1145 or less. LMICs have a GNI per capita between US $1146 and US $4515, while upper-middle-income countries fall between US $4516 and US $14,005. High-income countries are those with a GNI per capita exceeding US $14,005. Based on this classification, LMICs in the current review are defined as countries with GNI below US $14,005 per capita.)
- Studies that reported the implementation of digital health technologies in hospital settings (for both acute and chronic conditions).
- Studies that were reported between March 2020 and December 2023.
- Studies that were reported in the English language.
- Qualitative studies (phenomenology, ethnography, grounded theory, case study, etc), quantitative studies (case-control, cohort study, cross-sectional, randomized controlled trials, etc), mixed methods studies, and reviews (narrative review, scoping review, systematic review, meta-analysis, etc).
- Relevant gray literature (eg, Google Scholar).

**Exclusion criteria.**

- Studies that were nondigital-based (ie, studies that did not investigate the effectiveness of digital interventions, such as paper-based studies and postage surveys).
- Studies that implemented digital health technologies (DHTs) in primary care or community settings alone. However, studies that concurrently reported on the implementation of DHTs in hospital settings will still be included.
- Studies that used DHTs in dentistry and nonclinical medicine area (eg, dentistry, basic sciences, medical education, medical engineering, nutrition, dietetics, veterinary science, laboratory experimentations, and medical anthropology).

Systematic searches were conducted in PubMed, Scopus, and Web of Science databases. Potentially relevant gray literature was searched through targeted searches of Google Scholar. Lateral searching included screening reference lists in identified studies or reviews for relevant publications. Articles published in English between March 2020 and December 2023 were retrieved. Two investigators (SQY and NIHA) independently performed literature searches in the aforementioned electronic databases.

The search strategy was developed based on the “Population-Concept-Context” (PCC) framework as recommended by the Joanna Briggs Institute for Scoping Reviews [32] (Table 1). It aimed to identify the intersection between the “Population,” “Concept,” and “Context.” Based on the PCC framework, the search strategy was “Population combined” AND “Concept” AND “Context.” The details of the search strategy and search terms are tabulated in Multimedia Appendix 1.

**Table 1:** The PCC^a^ framework used to generate search terms.

Framework	Search terms
Population	Population 1: hospital settings (search terms as in Multimedia Appendix 1). Population 2: LMICsb (search terms as in Multimedia Appendix 1). Population combined: Population 1 AND Population 2.
*C*oncept	Digital health technologies (search terms as in Multimedia Appendix 1).
*C*ontext	Facilitators, barriers, and implementation.

^a^PCC: Population-Concept-Context.

^b^LMICs: lower- and middle-income countries

### Study Selection

Records were downloaded in Rayyan software (an artificial intelligence–assisted article screening software) [24] and after deduplication, all titles and abstracts were screened independently against the inclusion criteria by 2 reviewers (SQY and NIHA). Among all titles and abstracts found, 70 out of 279 (25%) were randomly selected and screened to establish interreviewer reliability. The interreviewer reliability (κ) was 0.63, indicating good reliability. Full-text articles of potentially relevant papers identified were screened independently by SQY and NIHA. Disagreements were resolved via discussion, with consultation from a third reviewer (BHC) if needed to reach a consensus. We included studies that met the review criteria and reported on barriers and facilitators of DHT implementation. For this review, DHTs were defined as a set of information and communications technologies used in medicine and health care to manage illnesses and to promote wellness [10].

### Charting the Data

Two authors (SQY and NIHA) independently extracted the following data from the included papers: authors, publication years, study objectives, study countries, disease conditions, types of DHTs, fields of clinical medicine where the DHTs are applied, study designs, sample sizes, characteristics of the study population, study location, and data collection methods of the intervention or program. We categorized the DHTs according to the types of equipment and method of operation. Any disagreements were resolved by discussion and consultation with a third author (BHC).

### Collating, Summarizing, and Reporting the Results

Descriptions of the included studies, such as study countries, types of DHTs, fields of clinical medicine where the DHTs are applied, and study designs were reported using descriptive statistics (eg, frequency distribution). Facilitators and barriers to DHT implementation, which were in the form of quantitative and qualitative data, were thematically analyzed using a deductive approach. To guide the thematic analysis, facilitators and barriers to DHT implementation were organized using the Practical, Robust Implementation, and Sustainability Model (PRISM) [33].

During the coding process, 2 authors (SQY and NIHA) identified the key components of the PRISM relevant to DHT implementation. They mapped the facilitators and barriers reported in the included studies to the predefined categories and subsequently grouped similar categories under overarching themes. By organizing the facilitators and barriers into themes and categories, patterns, trends, and relationships among the factors influencing DHT implementation can be identified, providing a structured framework for understanding the complexities of implementing DHTs in LMICs’ hospital settings [34].

### Consultation With Stakeholders

Clinicians and information technology (IT) experts from a local teaching hospital were invited to help interpret and contextualize the findings. Through interactive discussions and collaborative sessions, stakeholders provided valuable input on the categorization of facilitators and barriers as well as the implications of implementation of DHTs in their respective contexts. Moving forward, the authors plan to continue engagement with these stakeholders to disseminate the findings of the scoping review through presentations, policy briefs, and peer-reviewed publications.

## Results

### Literature Search

**Figure 1:**
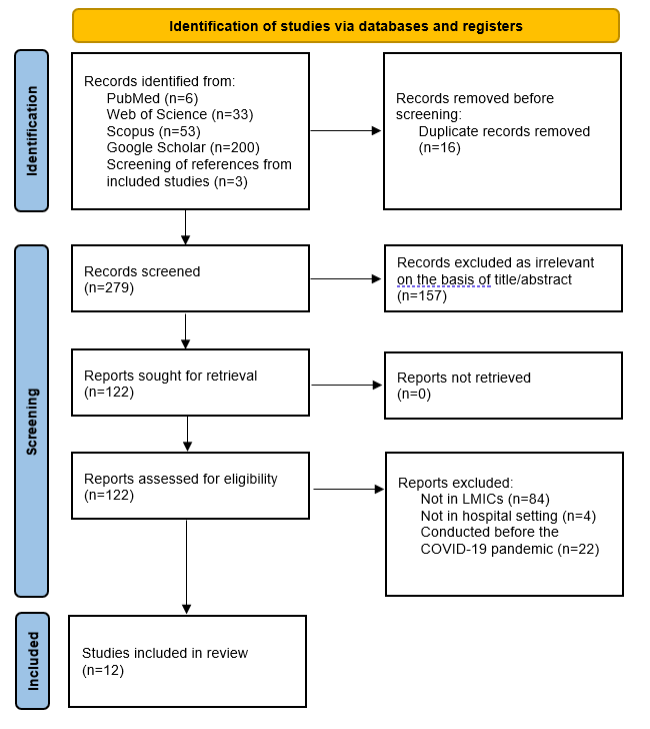
PRISMA (Preferred Reporting Items for Systematic Reviews and Meta-Analyses) flowchart. LMIC: lower- and middle-income country.

A systematic search yielded 295 titles and abstracts. After removing 16 duplicates, 279 unique articles remained. Among these, 157 were excluded based on title or abstract review. We thoroughly evaluated 122 full-text articles, and ultimately, 12 met the eligibility criteria. The PRISMA flow diagram was used to illustrate the search decision process of the scoping review [35] (Figure 1 and Multimedia Appendix 2).

### Characteristics of the Included Studies

Of the 12 articles, 6 (50%) were conducted in Asia [36-41], 4 (33%) in Africa [42-45], 1 (8%) in the Middle East [46], and 1 (8%) in South America [47]. Most of the studies (10/12, 83%) [36,37,40-47] did not specify the specific field of clinical medicine where DHTs were applied. However, some reported implementation in respiratory medicine (1/12, 8%) [38] and cancer (1/12, 8%) [39]. Telemedicine (5/12, 42%) [37,38,40,41,46] was the most commonly reported technology, followed by hospital information systems (4/12, 33%) [36,42,45,47], electronic medical records (2/12, 17%) [43,44], and mHealth (1/12, 8%) [39]. In terms of study design, there were 5 (42%) cross-sectional studies [37,41,42,44,46], 4 (33%) qualitative studies [38-40,43], 2 (17%) mixed method studies [45,47], and 1 (8%) case study [36]. The participant numbers ranged from 12 to 3386 individuals (Table 2).

**Table 2:** Overview of the characteristics of the included studies.

Authors and publication years	Study details								
	Objective(s)	Country	Disease condition	Types of DHTs^a^	Field of clinical medicine	Design	Sample sizes and characteristics of the population	Location	Data collection method
Abdulai et al [36]	To investigate the attitudes, opportunities, and challenges in using health information system.	India	General	Health information system	General	Case study	20 health care providers and managers	Apollo Hospital and Medanta Hospital	Self-administered questionnaire
Alboraie et al [37]	To assess the usefulness of telemedicine and the different barriers hindering its utilization.	Egypt	General	Telemedicine	General medicine, surgery, and radiology	Cross-sectional study	642 health care providers	All hospitals across Egypt	Online self-administered questionnaire
Baradwan and Al-Hanawi [46]	To gain a holistic understanding of the perceptions and barriers of the end users (participants) toward the utility of telemedicine.	Saudi Arabia	General	Telemedicine	General	Cross-sectional study	1024 individuals	Nationwide	Self-administered questionnaire
Jiang et al [38]	To explore the perceptions and experiences of older patients and health care providers in the application of telehealth and online health information to chronic disease management of chronic obstructive pulmonary disease.	China	Chronic obstructive pulmonary disease	Telemedicine	Respiratory medicine	Qualitative	31 older patients with chronic obstructive pulmonary disease and 23 health care providers	A community hospital in Jiangnan, China	In-depth interviews
Mekuria et al [42]	To assess the level of health information system utilization among health professionals in public health facilities.	Ethiopia	General	Health information system	General	Cross-sectional study	378 health care providers	Dire Dawa Administration in eastern Ethiopia	Self-administered questionnaire
Mussi et al [47]	To understand the implementation of a hospital information system in university hospitals.	Brazil	General	Health information system	General	Exploratory mixed methods	770 health managers (24 in in-depth interviews, 10 in focus group discussions, and 736 in cross-sectional study)	5 university hospitals in Brazil	Interviews, focus group discussions, questionnaires, and documentary research
Ngugi et al [43]	To explore end users’ perceptions and experiences on factors facilitating and hindering electronic medical record use in health care facilities.	Kenya	General	Electronic medical record	General	Qualitative	20 health care providers	20 health care facilities in Kenya	Focus group discussion
Ning et al [39]	To explore the needs and perceptions of patients with head and neck cancer regarding mobile health–based physical activity programs.	China	Head and neck cancer	Mobile health	ENT^b^	Qualitative	17 patients diagnosed with head and neck cancer	First Hospital of Shanxi Medical University, Taiyuan, China	In-depth interviews
Shardha et al [40]	To explore the view of health care professionals regarding the benefits, challenges, and prospects of telemedicine to address the gap that hinders its effective use in the rural areas.	Pakistan	General	Telemedicine	Nonsurgical disciplines	Qualitative (narrative)	12 health care providers	Two tertiary hospitals in Sindh, Pakistan	In-depth interviews
Tesfa et al [44]	To assess the level of electronic health record utilization and associated factors among health care professionals at teaching hospitals.	Ethiopia	General	Electronic medical record	General	Cross-sectional study	383 health care providers	University of Gondar Specialized Teaching Hospital and Tibebe Ghion Specialized Teaching Hospital	Self-administered questionnaire
Tilahun et al [45]	To evaluate the outcomes and share experiences of working with universities to strengthen the national health information system.	Ethiopia	General	Health information system	General	Mixed methods study	23 health care providers	47 health care organizations in Ethiopia	In-depth interviews
Yu-Tong et al [41]	To assess telehealth readiness among clinical nurses and explore the factors that affect their telehealth readiness.	China	General	Telemedicine	General	Cross-sectional study	3386 nurses	19 hospitals in China	Self-administered questionnaire

^a^DHT: digital health technology.

^b^ENT: ear, nose, and throat.

### Facilitators and Barriers to DHT Implementation

Among the 12 studies, 9 explored both the facilitators and barriers to implementing DHTs in hospital settings in LMICs since the onset of COVID-19 [36-40,43-45,47]. Two studies focused solely on facilitators [41,42], while 1 solely addressed barriers [46]. A total of 63 facilitators and 44 barriers were identified. Subsequently, these facilitators and barriers were systematically organized into 6 themes according to the PRISM, which include the following: (1) Aspects of the Health Care System; (2) Perspectives of Patients; (3) External Environment; (4) Implementation of Sustainable Infrastructure; (5) Characteristics of Health Care Organization; and (6) Characteristics of Patients. These are outlined in Table 3.

**Table 3:** Facilitators and barriers to DHT^a^ implementation in hospital settings in lower- and middle-income countries after the COVID-19 pandemic.

Themes and categories		Codes for facilitators	Codes for barriers
**Aspects of the Health Care System (n^b^=11)**			
	Readiness (n_f_^c^=4; n_b_^d^=3)	Understand the basics of information technology [37] Knowledge in protecting data confidentiality [37] High level of self-efficacy [42] Good attitude toward DHTs [42] High awareness about the implementation of DHTs [47] High personal interest and motivation [47] The high willingness of health care providers to provide DHTs for patients [41]	Inadequate awareness regarding DHTs [40,44] Feeling that DHTs are only complementary to traditional health care [38] Preference of some health care providers for printed materials [44]
	Strength of the evidence base (n_f_=1; n_b_=2)	The ability of DHTs to offer statistical analysis and reports for decision-making [36] The ability of DHTs to offer timely and up-to-date patient information [36]	Concerns regarding accuracy in disease diagnosis and treatment [40,46]
	Addresses barriers to frontline staff (n_f_=1; n_b_=2)	Easy access to computers in the working area [44]	Fear of inappropriate data protection might breach patient’s privacy [37] Inadequate computers at the workplace and service point local area network at the workplace [43]
	Coordination across departments and specialties (n_f_=1; n_b_=1)	Use of single information technology policy and standard hospital information system [47]	Lack of interoperability standards between systems [47]
	Burden (complexity and cost; n_f_=4; n_b_=1)	Perceive that operations of DHTs are not complex [42] The availability of information technology budget [47] Regular upgrades on DHT hardware [43] Funding support focusing on research and learning opportunities on DHTs [45]	The need for retrospective data entry into systems [43]
	Usability and adaptability (n_f_=4; n_b_=3)	The ability of DHTs to prevent cross-infection [38,40] Ability to provide health care in remote areas [40] The functionalities of DHTs align with hospital’s practices [47] User-friendliness of DHTs [43]	Difficulty in performing physical examination with DHTs [40] Lack of compliance among health care professionals and patients [40] DHTs might reduce the quality of medical services by increasing the probability of medical mistakes [37] Perceive DHTs as research agenda only [45] Some DHTs are not user-friendly [36]
	Trialability and reversibility (n_f_=1; n_b_=0)	Conduct beta tests and pilot tests before implementation of DHTs [47]	N/Ae
	Ability to observe results (n_f_=3; n_b_=0)	Positive previous experiences using DHTs [41,47] The ability of DHTs to increase administrative and health care efficiency [36] The ability of DHTs to reduce medication errors [36]	N/A
**Perspectives of Patients (n=5)**			
	Patient centeredness (n_f_=1; n_b_=1)	Using technology gives a superior feeling [38] Repeated motivational or praising words increase patients’ usage of DHTs [38]	The feeling of the distance between the patient and health care providers during remote interaction [38] The feeling of being forced to use DHTs [38]
	Addresses patient barriers (n_f_=1; n_b_=1)	Involvement of patients’ children could improve the efficiency and effectiveness of DHTs [38]	Patients’ resistance to change practice [46]
	Seamlessness of transition between program elements (n_f_=1; n_b_=0)	Unification of hospitals in a single network and centralized management [47]	N/A
	Service and access (n_f_=1; n_b_=0)	Easy access to online health information and applying the knowledge [38]	N/A
	Burden (complexity and cost; n_f_=2; n_b_=0)	The ability of DHTs to reduce stress among patients [40] DHTs are cost-effective [38]	N/A
	Feedback of results (n_f_=2; n_b_=0)	DHTs are easy and convenient to use [38,39] DHTs are able to improve the comfort level of patients [39]	N/A
**External Environment (n=3)**			
	Competition (n_f_=0; n_b_=2)	N/A	Competition for resources between information technology equipment and other health care equipment [45,47]
	Regulatory environment (n_f_=1; n_b_=2)	Good communication process between the regulatory authority and the hospitals [47]	Political and economic instabilities [45,47]
	Community resources (n_f_=1; n_b_=0)	Good internet access among users of DHTs [44]	N/A
**Implementation of Sustainable Infrastructure (n=5)**			
	Adopter training and support (n_f_=1; n_b_=0)	Familiarity with handling DHT tools [40]	N/A
	Relationship and communication with adopters (n_f_=0; n_b_=1)	N/A	The communication gap between supervisors and supervisees [45]
	Adaptable protocols and procedures (n_f_=1; n_b_=0)	Remote health service can be carried out easily among clinicians [37]	N/A
	Facilitation of sharing of best practices (n_f_=1; n_b_=0)	Experience sharing between advanced and beginner hospitals [47]	N/A
	Plan for sustainability (n_f_=1; n_b_=1)	Health care facilities prioritize the implementation and sustainability of DHTs [47]	The end of the COVID-19 pandemic reduced the demand for DHTs [46]
**Characteristics of the Health Care Organization (n=11)**			
	Organizational health and culture (n_f_=3; n_b_=1)	The availability of fair-to-good organizational support [42] Commitment from the top management or managers [47] Top management mandates the adoption of DHTs [47] Promote experience and knowledge sharing among hospital networks [47] A workplace culture that is receptive to change [47] Understand the policies made by the government [41]	Frequent changes in the hospital management team [47] Lack of medium- and long-term information technology policy planning [47]
	Management support and communication (n_f_=2; n_b_=1)	Presence of supervision at the workplace [42] The availability of fair-to-good technical support [42] Provision of support to groups with little knowledge of technology [47]	Lack of planning and implementation of system design [47]
	Shared goals and cooperation (n_f_=2; n_b_=1)	The information technology department of the hospital is directly linked to local information technology sectors [47] Create a multidisciplinary management committee [47] Partnership with universities to develop DHTs [45]	Health care providers’ resistance to change practice [46]
	Clinical leadership (n_f_=1; n_b_=1)	High level of decision-making autonomy in the workplace [42]	Weak leadership and poor commitment at the hospital level [45]
	Systems and training (n_f_=3; n_b_=5)	The workplace has sufficient facilities [37] The availability of continuous on-the-job training [37,43,47] Regular system upgrades [43] The availability of prompt technical assistance from desk support [43]	Lack of training in advanced technologies [36,40] Delays in system development, implementation, and updates [43,47] Frequent power blackouts [43] Poor electrical power and backup facilities [36] Lack of skill among health care providers in accessing and using DHTs [44]
	Data and decision support (n_f_=2; n_b_=2)	The availability of 24-hour technical support services at the workplace [37] Centralization of system development and technical support [47]	Lack of contingency plan for DHTs [47] Delayed information technology support [43]
	Staffing and incentives (n_f_=3; n_b_=3)	Workplace has sufficient trained personnel in DHTs [37] Improved financing of human and material resources [47] Sufficient human resource allocation [41]	Lack of incentives for health care providers to use DHTs [38] High staff turnover rate, work overload, and reduced staffing [43,47] Health care providers who are old aged [43]
	Expectation of sustainability(n_f_=0; n_b_=3)	N/A	Health care providers do not have sufficient time and energy to participate and offer timely feedback while using DHTs [38,44] Unrealistic expectations toward DHTs [47]
**Characteristics of patients (n=4)**			
	Demographics (n_f_=0; n_b_=2)	N/A	Older patients contemplated using and have low confidence in DHTs [38] Personal inertia and the conservative mentality of the older generation [38] Costs of accessing the internet beyond affordability for patients from lower socioeconomic status [39] Poor cellular network connectivity and internet access in some geographical areas [39]
	Knowledge and beliefs (n_f_=2; n_b_=4)	Confidence to learn, engage, and sustainably participate in DHTs [38] High eHealth and computer literacy among patients [38,44] High information-searching skills among patients [44]	Patients have poor digital literacy and skills in DHTs [36,38,39] Patients have skepticism about the accuracy of remote diagnosis [38] Social prejudice against DHTs [38]

^a^DHT: digital health technology.

^b^n: number of studies in general.

^c^n_f_: number of studies that reported on facilitators.

^d^n_b_: number of studies that reported on barriers.

^e^N/A: not applicable.

Specifically, the theme of Aspects of the Health Care System included 23 facilitators and 13 barriers (n=11 studies). Among the prominent facilitators were the ability of DHTs to prevent cross-infection (n=2) and positive previous experiences using DHTs (n=2). By contrast, inadequate awareness regarding DHTs (n=2) and concerns regarding accuracy in disease diagnosis and treatment (n=2) were the leading barriers highlighted.

Regarding the theme of Perspectives of Patients, 9 facilitators and 3 barriers were reported (n=5 studies). Among these, the convenience and easiness of using DHTs (n=2) were noted as the primary facilitator. However, the feeling of distance between the patient and health care providers during remote interaction (n=1), the feeling of being forced to use DHTs (n=1), and patients’ resistance to change practice could pose challenges to the implementation of DHTs in hospital settings within LMICs.

The successfulness of implementing DHTs also relies on the theme of External Environment (n=3 studies). Within this context, 2 facilitators are aiding the implementation, which include a good communication process between the regulatory authority and the hospitals (n=1) and good internet access among users of DHTs (n=1). Among the 2 barriers identified in this context, competition for resources between IT equipment and other health care equipment (n=2) stands out as the more prevalent barrier.

The theme of Implementation of Sustainable Infrastructure comprises 4 facilitators and 2 barriers (n=5 studies). Notably, familiarity with handling DHTs tools (n=1), the ease of conducting remote health service among clinicians (n=1), and experience sharing between advanced and beginner hospitals (n=1) emerge as facilitators for DHT implementation. Conversely, barriers in this theme include the communication gap between stakeholders (n=1) and the end of the COVID-19 pandemic reducing the demand for DHTs (n=1).

The Characteristics of Health Care Organization theme consists of 22 facilitators and 17 barriers to DHT implementation (n=11 studies). Previous studies have highlighted the availability of continuous on-the-job training (n=3) as the most frequent facilitator of DHT implementation in the health care organization. Conversely, barriers such as lack of trainings in advanced technologies (n=2); delays in system development, implementation, and updates (n=2); high staff turnover rate, work overload, and reduced staffing (n=2); as well as insufficient time and energy among health care providers to participate and offer timely feedback while using DHTs (n=2) prevent the implementation of DHTs.

The Characteristics of Patients was the final theme that could influence the successfulness of DHT implementation in LMICs’ hospital settings. It comprised 3 facilitators and 7 barriers (n=4 studies). Notably, the most frequently mentioned facilitator in this theme is the high eHealth and computer literacy among patients (n=2). Not surprisingly, the exact opposite, that is, patients with poor digital literacy and skills in DHTs (n=3), is reported as the commonest barrier.

**Figure 2:**
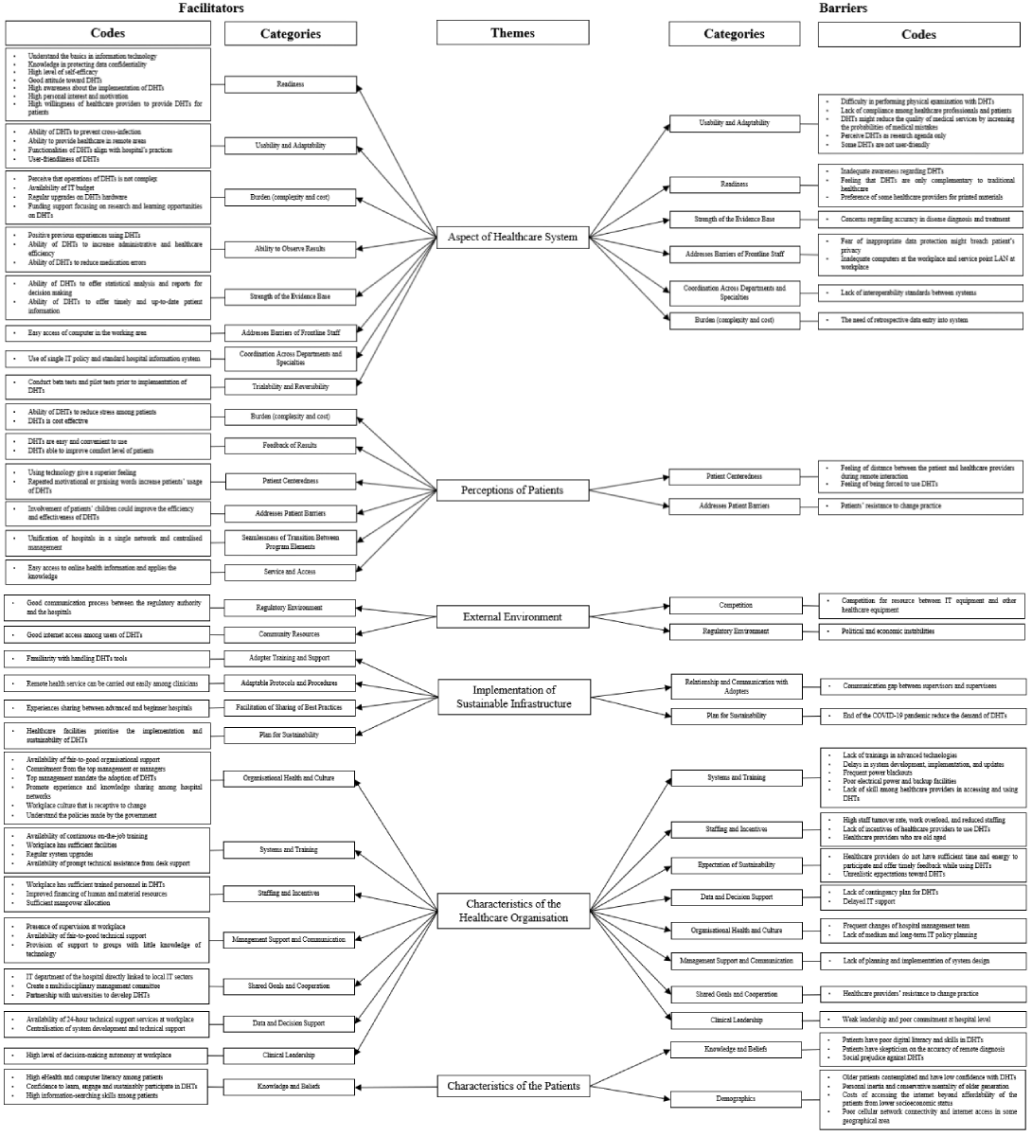
Summary of the codes, categories, and themes of facilitators and barriers of digital health technology (DHT) implementation. IT: information technology; LAN: local area network.

The relationship between codes, categories, and themes of facilitators and barriers to DHT implementation is summarized in Figure 2, in which the codes and categories are arranged from the most frequent (top) to the least frequent (bottom).

**Figure 3:**
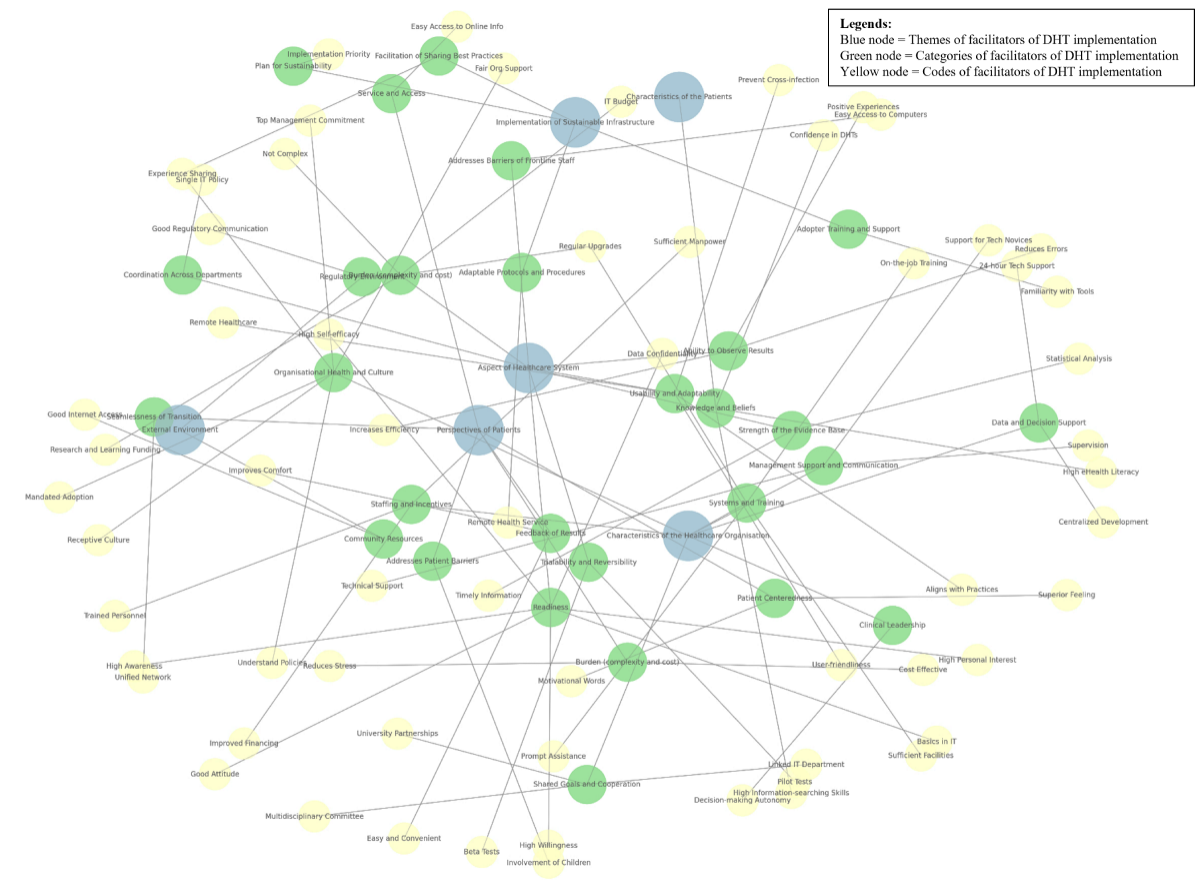
Network analysis to illustrate the relationship between the codes of facilitators of digital health technologies (DHTs) implementation in lower- and middle-income countries' (LMICs) hospital settings since the onset of the COVID-19 pandemic.

**Figure 4:**
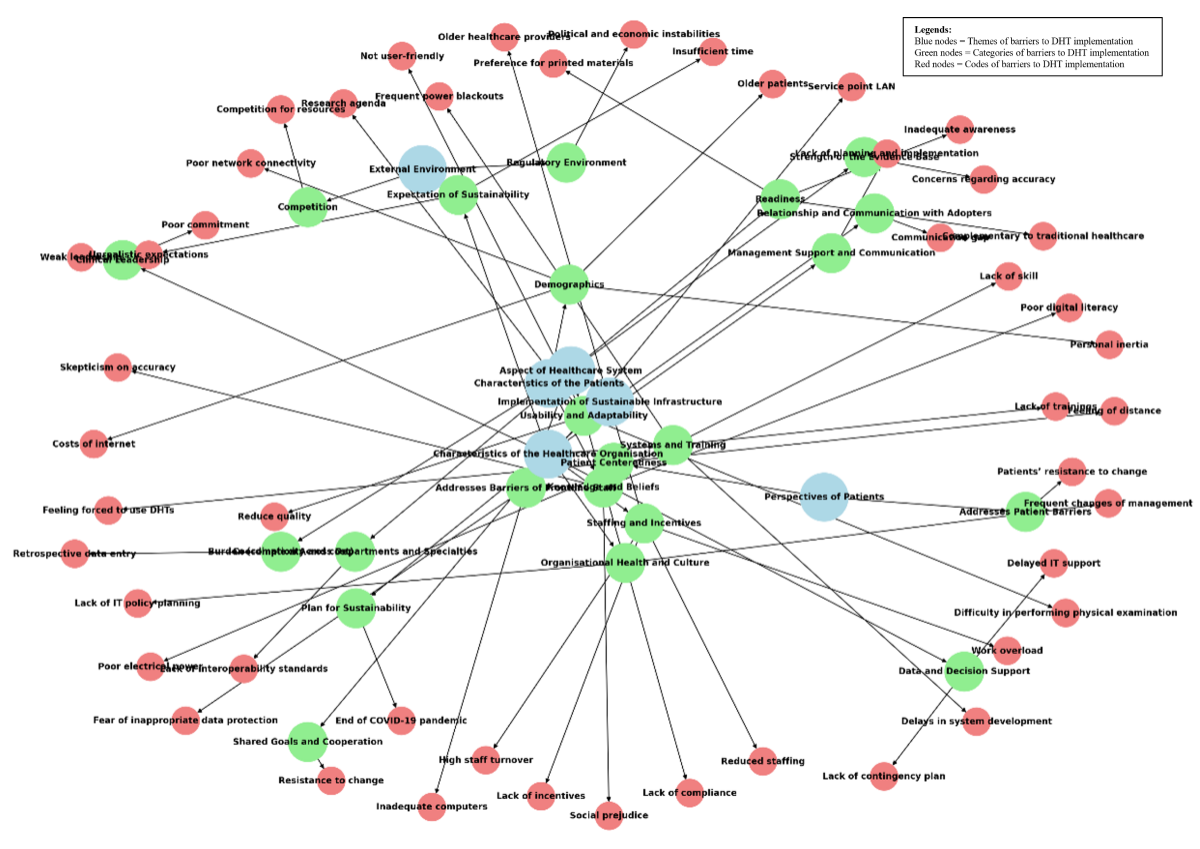
Network analysis to illustrate the relationship between the codes of barriers of digital health technologies (DHTs) implementation in lower- and middle-income countries' (LMICs') hospital settings in the post-COVID-19 era. IT: information technology; LAN: local area network.

Figure 3 illustrates the network analysis of the facilitators of DHT implementation. Of note, facilitators such as “Perspectives of Patients” and “Aspect of the Health Care System” are central to the network. This suggests their broad impact across multiple domains, making them key intervention points for DHT implementation. Although no obvious clusters were observed, the interconnectedness suggests that addressing certain facilitators (eg, “Perspectives of Patients,” “Aspect of the Health Care System,” and “Characteristics of the Health Care Organization”) can have ripple effects on others. Figure 4 illustrates the network analysis of the barriers to DHT implementation. It was noted that some barriers are more central, such as the “Characteristics of Health Care Organizations,” “Aspect of the Health Care System,” “Implementation of Sustainable Infrastructure,” and “Characteristics of Patients” barriers, highlighting their pivotal roles in the network. These central barriers act as key influencers and bridge multiple barriers. Meanwhile, the “External Environment” and “Perspectives of Patients” barriers are more toward the periphery and, hence, have less impact on the network. A cluster was observable in the network analysis, involving the “Characteristics of Health Care Organization” and “Aspect of the Health Care System” barriers. Many barriers are interconnected, which indicate that addressing one (eg, “Characteristics of Health Care Organizations”) may alleviate others. However, it should be noted that some nodes, such as “Readiness” and “Usability and Adaptability,” can be both facilitators of and barriers to DHT implementation, and hence, can appear in both network analyses.

**Figure 5:**
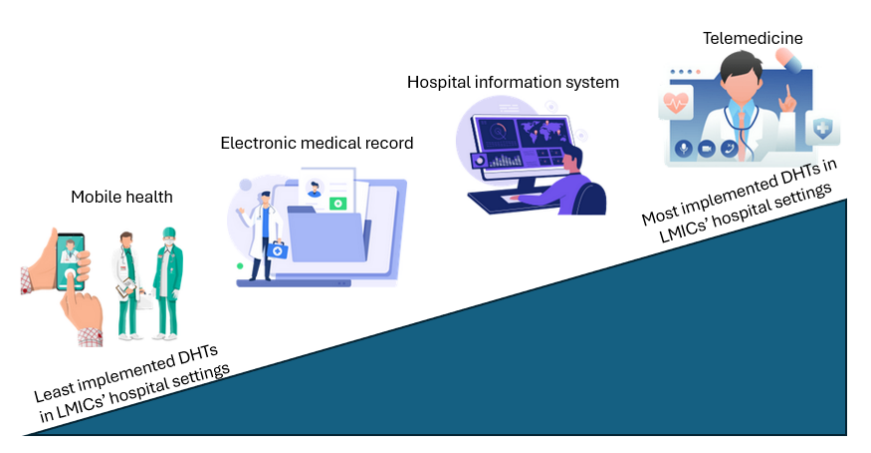
Digital health technologies (DHTs) in the lower- and middle-income countries (LMICs) since the onset of the COVID-19 pandemic.

Since the onset of the COVID-19 pandemic, the most commonly utilized DHTs in LMICs’ hospitals were telemedicine, followed by health information systems, electronic medical records, and mHealth, as depicted in Figure 5.

## Discussion

### Facilitators of and Barriers to DHT Implementation in LMICs

The most common facilitators of DHT implementation include the convenience and easiness of using DHTs, the ability of DHTs to prevent cross-infection, positive previous experiences using DHTs, the availability of continuous on-the-job training, and high eHealth and computer literacy among patients. By contrast, we identified that the most frequently reported barriers are inadequate awareness regarding DHTs; concerns regarding accuracy in disease diagnosis and treatment; competition for resources between IT equipment and other health care equipment; lack of trainings in advanced technologies; delays in system development, implementation, and updates; high staff turnover rate, work overload, and reduced staffing; insufficient time and energy among health care providers to use DHTs; and patients with poor digital literacy and skills in DHTs.

To our knowledge, this scoping review is the first to examine the facilitators and barriers to implementing DHTs in hospitals within LMICs since the onset of the COVID-19 pandemic. It is important to note the scarcity of published literature on this topic in LMICs. Even existing reviews often focused on studies from high-income countries or concentrated on specific diseases [27,48,49]. For example, a systematic review by Whitelaw et al [27], comprising 29 studies, investigated DHT implementation in cardiovascular diseases predominantly in high-income countries. As expected, the facilitators and barriers identified in this review significantly differed from our findings. Whitelaw et al highlighted facilitators such as enhanced communication with clinicians, personalized DHT components, user-friendly interfaces, institutional and organizational support, increased efficiency, and perceived usefulness of DHTs. The reported barriers were complex technology, technological apprehension, increased workload, unreliable technology, and lack of integration with electronic medical records. These discrepancies may stem from various factors such as differences in health care infrastructure [50], socioeconomic contexts [51], health care financing [52], as well as costs of DHT development and implementation [53] between high-income and LMIC settings. Additionally, the focus on specific diseases in previous literature might not fully capture the diverse facilitators and barriers relevant to DHT implementation across various health care contexts.

### DHTs That Have Been Implemented

To our surprise, telemedicine was the most commonly reported technology implemented in hospital settings since the onset of the COVID-19 pandemic, exceeding other DHTs such as hospital information systems, electronic medical records, and mHealth. This can be understood by the fact that telemedicine is well-suited for health care providers and patients who are self-isolating, as it effectively reduces the risk of COVID-19 transmission. It eliminates the need for direct physical contact, ensures ongoing care for the community, and ultimately lowers the rates of illness and death during the COVID-19 pandemic [54].

### Classification Framework for DHT Implementation in LMICs

**Figure 6:**
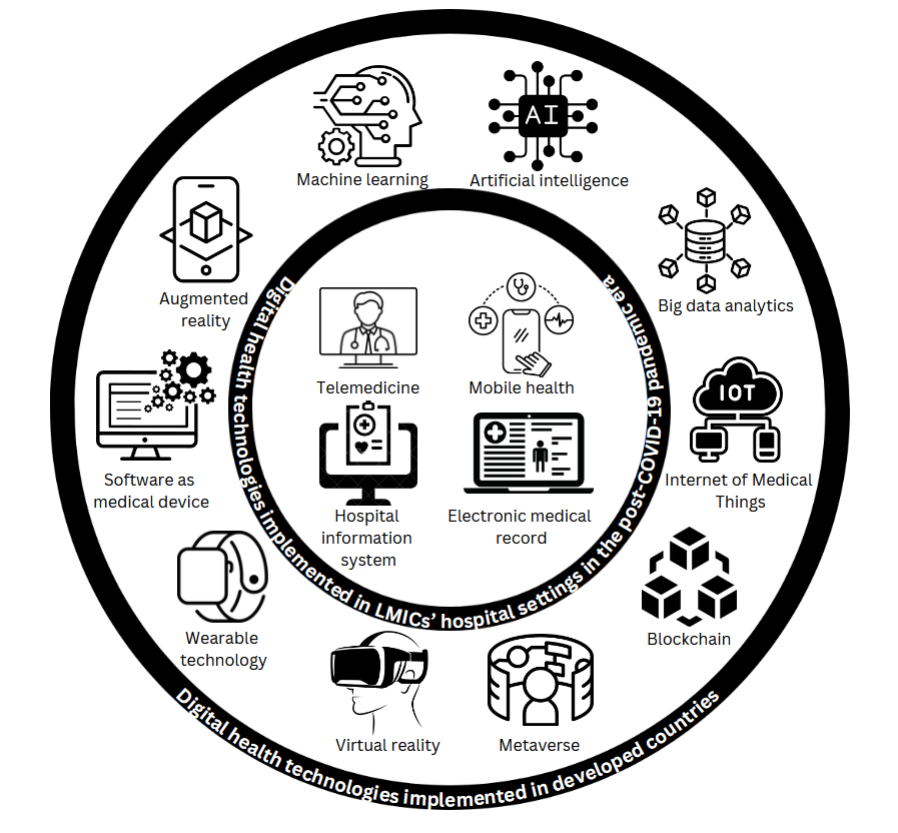
Classification framework of digital health technologies (DHTs). LMIC: lower- and middle-income country.

Despite the emergence of various innovative technologies such as wearable technologies, metaverse, Internet of Medical Things, blockchain in health care, big data analytics, artificial intelligence, machine learning, software as medical devices, augmented reality, and virtual reality, the implementation of DHTs in hospitals within LMICs remains rudimentary compared with high-income countries. The current utilization of DHTs in LMICs, such as telemedicine, hospital information systems, electronic medical records, and mHealth, has informed the key components of a new classification framework that emphasizes LMICs. Based on recent literature [13,15,17-23,55] and the specific facilitators and barriers to utilizing these DHTs in LMICs, we propose a classification framework of DHTs, as illustrated in Figure 6.

This classification framework is justified by the fact that simpler DHTs, such as telemedicine, hospital information systems, electronic medical records, and mHealth, are easier to implement in LMICs due to lower costs and infrastructure requirements, adaptability to limited resources, and minimal training needs. These technologies rely mainly on basic internet connectivity, mobile devices, and computers, making them more accessible and feasible given the broader availability of mobile networks in LMICs. They also integrate easily into existing health care workflows, requiring only short training sessions and basic digital literacy. Furthermore, because LMICs prioritize addressing immediate health care needs such as infectious diseases, these accessible technologies receive more support and funding. By contrast, advanced technologies such as artificial intelligence, virtual reality, and blockchain demand specialized infrastructure, technical expertise, and substantial financial resources, which are often scarce in LMICs.

This proposed classification framework also consists of other DHTs that were implemented in high-income countries. This, hopefully, will aid health care stakeholders in LMICs to delineate the scope of DHTs in LMICs and help them to prioritize the right DHTs that could be implemented in the scarcity of resources. Consequently, policy makers can allocate health care funding (eg, the Ministry of Health can determine the DHT classes that are eligible for subsidies), and insurers can reimburse patients who utilized DHTs (ie, identifying which DHT classes can be covered by insurance policies) [56,57]. Moreover, clear and effective communication among the public, health care providers, and technology experts can be facilitated [57,58]. Legally, a defined classification framework enables authorities to regulate and govern DHT usage [58,59]. Finally, a clearly outlined classification framework for DHTs may streamline implementation, especially in resource-scarce environments, as stakeholders can prioritize which DHT classes should be implemented [56].

### Strengths and Limitations

This scoping review possesses several notable strengths. Initially, we comprehensively gathered data from a diverse array of databases, including gray literature sources such as Google Scholar. This deliberate choice was made to ensure the inclusivity and robustness of the studies included. Additionally, during the planning phase of the review, we actively engaged relevant stakeholders, including information technologists, seeking and incorporating their feedback into the review protocol. Furthermore, in structuring and categorizing the identified barriers and facilitators, we utilized a conceptual model developed by Feldstein and Glasgow [33], thereby enhancing the analytical rigor of the review. Moreover, our review offers a wealth of qualitative and quantitative insights into the implementation of DHTs, providing comprehensive guidance for stakeholders interested in implementing DHTs within their respective settings.

Despite its strengths, our review does have certain limitations. Specifically, in focusing on identifying barriers and facilitators of DHT implementation in hospital settings within LMICs, we restricted our inclusion criteria to studies conducted solely within hospitals. Consequently, many studies providing insights from primary care and community perspectives were excluded. Additionally, studies that did not explicitly address facilitators and barriers were omitted, potentially overlooking valuable information on other beneficial DHTs for patient care. Furthermore, as our primary aim was to offer an overview of existing literature on DHTs, we did not apply any quality control measures to the included studies. The variation in analytical methods, interview questions, and study populations across the included papers presents a potential inconsistency in the findings and makes quality assessment difficult. Additionally, with 128 LMICs at the time of the literature search, listing each country by name in the search string was impractical, which could result in some LMICs not being captured in the search results.

### Implications and Recommendations

Understanding the facilitators and barriers to DHTs in hospital settings of LMICs has profound implications for both clinical practice and research. Identifying facilitators of DHT implementation enables the effective integration of DHTs in hospitals, leading to improved diagnostic accuracy, better patient monitoring, improved patient records management, and the availability of remote health care services. Conversely, recognizing the barriers to DHT implementation can prompt various stakeholders in LMICs to devise strategies that address issues in terms of financial resources, technological infrastructure, and knowledge among health care providers. In terms of research, this review is hoped to promote international collaborations, governmental support, and investment in digital infrastructure, which in turn boost the capacity for data collection, analysis, and sharing. These advancements can enhance research capabilities, allowing for more comprehensive epidemiological studies, real-time disease monitoring, and the development of context-specific health interventions.

To maximize the benefits of DHTs in hospital settings in LMICs, several recommendations can be made to leverage facilitators and mitigate barriers. First, the convenience and ease of using DHTs should be emphasized through user-friendly interfaces and intuitive designs, ensuring that health care providers can quickly and efficiently adopt these technologies in their daily routines. Second, the role of DHTs in preventing cross-infection should be highlighted in training programs and awareness campaigns, particularly in regions where infectious diseases are prevalent. Positive previous experiences with DHTs should be shared widely to build trust and demonstrate the tangible benefits of these technologies. Continuous on-the-job training must be provided to keep health care workers up-to-date with the latest advancements and best practices, while also enhancing their technical proficiency. Promoting high eHealth and computer literacy among patients through community outreach and education programs will further support the effective use of DHTs.

Addressing barriers requires a multifaceted approach. To tackle inadequate awareness and concerns regarding the accuracy of DHTs, it is crucial to implement comprehensive educational initiatives that highlight the reliability and clinical efficacy of these technologies. Clear, evidence-based information should be disseminated to health care providers and patients to build confidence in DHTs. Competition for resources between IT equipment and other health care tools can be mitigated by advocating for balanced budget allocations and seeking external funding or partnerships to supplement resources. Training programs in advanced technologies must be expanded to equip health care providers with the necessary skills to use DHTs effectively. To address delays in system development and implementation, streamlined processes and timelines should be established, with dedicated project management teams overseeing these initiatives. High staff turnover rates and work overload can be managed by improving working conditions, offering competitive salaries, and providing career development opportunities. Finally, enhancing digital literacy among patients through targeted educational programs will ensure that they can engage with DHTs effectively, ultimately leading to better health outcomes. By implementing these recommendations, LMICs can overcome barriers and fully harness the potential of DHTs to improve clinical practice and advance health care research.

### Conclusions

The scoping review on DHT implementation in LMICs since the onset of the COVID-19 pandemic underscores significant policy, clinical, and research implications. Policy makers can utilize the insights to craft targeted strategies for DHT adoption, while the developed classification framework aids in prioritizing DHTs. In clinical settings, understanding DHT types, facilitators, and barriers enhances decision-making for improved patient care and resource optimization. Additionally, investing in capacity building and training programs for health care professionals is crucial, with a focus on enhancing digital literacy and technical skills. Moreover, identifying research priorities and aligning funding with key challenges such as improving digital literacy and addressing concerns about DHT accuracy will drive advancements in DHT implementation and health care delivery in LMICs.

In the postpandemic era, telemedicine has been widely used in hospital settings in LMICs, among other DHTs. Our findings also reveal numerous facilitators and barriers to DHT implementation in LMIC hospital settings. These factors can be organized into 6 themes, namely, (1) Aspects of the Health Care System; (2) Perspectives of Patients; (3) External Environment; (4) Implementation of Sustainable Infrastructure; (5) Characteristics of Health Care Organization; and (6) Characteristics of Patients.

## Appendix

Multimedia Appendix 1 Search strategies.

Multimedia Appendix 2 The PRISMA-ScR (Preferred Reporting Items for Systematic Reviews and Meta-Analyses Extension for Scoping Reviews) checklist.

## Abbreviations

DHT: digital health technology
GNI: gross national income
IT: information technology
LMIC: lower- and middle-income country
mHealth: mobile health
PCC: Population-Concept-Context
PRISM: Practical, Robust Implementation and Sustainability Model
PRISMA-ScR: Preferred Reporting Items for Systematic Reviews and Meta-Analyses Extension for Scoping Reviews
WHO: World Health Organization

## Data Availability

All data retrieved and analyzed are included in this scoping review.

## References

[1] World Health Organization (WHO) (2023). Tracking universal health coverage: 2023 global monitoring report. WHO.

[2] Shah SA, Safian N, Ahmad S, Nurumal SR, Mohammad Z, Mansor J, Wan Ibadullah WAH, Shobugawa Y, Rosenberg M (2021). Unmet healthcare needs among elderly Malaysians. JMDH.

[3] Njagi Purity, Arsenijevic Jelena, Groot Wim (2020). Cost-related unmet need for healthcare services in Kenya. BMC Health Serv Res.

[4] Sohn M, Che X, Park Hee-Jung (2020). Unmet healthcare needs, catastrophic health expenditure, and health in South Korea's universal healthcare system: progression towards improving equity by NHI type and income level. Healthcare (Basel).

[5] Baek Suyon, Choi Eun-Hi, Lee Jungeun (2020). Unmet healthcare needs of children in vulnerable families in South Korea: finding from the Community Child Center Child Panel Survey. Int J Environ Res Public Health.

[6] Kim Jungah, You Myoungsoon, Shon Changwoo (2021). Impact of the COVID-19 pandemic on unmet healthcare needs in Seoul, South Korea: a cross-sectional study. BMJ Open.

[7] Harris Ricci B, Cormack Donna M, Stanley James (2019). Experience of racism and associations with unmet need and healthcare satisfaction: the 2011/12 adult New Zealand Health Survey. Aust N Z J Public Health.

[8] Rosenthal A, Waitzberg R (2023). The challenges brought by the COVID-19 pandemic to health systems exposed pre-existing gaps. Health Policy Open.

[9] Mitgang EA, Blaya JA, Chopra M (2021). Digital health in response to COVID-19 in low- and middle-income countries: opportunities and challenges. Glob Policy.

[10] Ronquillo Y, Meyers A, Korvek S (2025). Digital Health. StatPearls.

[11] Istepanian Robert S H (2022). Mobile health (m-Health) in retrospect: the known unknowns. Int J Environ Res Public Health.

[12] Roy Joy, Levy Deborah R, Senathirajah Yalini (2022). Defining telehealth for research, implementation, and equity. J Med Internet Res.

[13] Huhn Sophie, Axt Miriam, Gunga Hanns-Christian, Maggioni Martina Anna, Munga Stephen, Obor David, Sié Ali, Boudo Valentin, Bunker Aditi, Sauerborn Rainer, Bärnighausen Till, Barteit Sandra (2022). The impact of wearable technologies in health research: scoping review. JMIR Mhealth Uhealth.

[14] Ibrahim Ariff Azfarahim, Ahmad Zamzuri Mohd 'Ammar Ihsan, Ismail Rosnah, Ariffin Ahmad Husni, Ismail Aniza, Muhamad Hasani Muhamad Hazizi, Abdul Manaf Mohd Rizal (2022). The role of electronic medical records in improving health care quality: a quasi-experimental study. Medicine (Baltimore).

[15] Belle Ashwin, Thiagarajan Raghuram, Soroushmehr S M Reza, Navidi Fatemeh, Beard Daniel A, Najarian Kayvan (2015). Big data analytics in healthcare. Biomed Res Int.

[16] Dwivedi Ruby, Mehrotra Divya, Chandra Shaleen (2022). Potential of Internet of Medical Things (IoMT) applications in building a smart healthcare system: a systematic review. J Oral Biol Craniofac Res.

[17] Yoon Hyung-Jin (2019). Blockchain technology and healthcare. Healthc Inform Res.

[18] Petrigna Luca, Musumeci Giuseppe (2022). The metaverse: a new challenge for the healthcare system: a scoping review. J Funct Morphol Kinesiol.

[19] Yu J, Zhang J, Sengoku S (2023). Innovation process and industrial system of US Food and Drug Administration–approved software as a medical device: review and content analysis. J Med Internet Res.

[20] Eckert Martin, Volmerg Julia S, Friedrich Christoph M (2019). Augmented reality in medicine: systematic and bibliographic review. JMIR Mhealth Uhealth.

[21] Halbig  A, Babu  Sk, Gatter  S, Latoschik  Me, Brukamp K, von Mammen  S (2022). Opportunities and challenges of virtual reality in healthcare – a domain experts inquiry. Front Virtual Real.

[22] Xu Yongjun, Liu Xin, Cao Xin, Huang Changping, Liu Enke, Qian Sen, Liu Xingchen, Wu Yanjun, Dong Fengliang, Qiu Cheng-Wei, Qiu Junjun, Hua Keqin, Su Wentao, Wu Jian, Xu Huiyu, Han Yong, Fu Chenguang, Yin Zhigang, Liu Miao, Roepman Ronald, Dietmann Sabine, Virta Marko, Kengara Fredrick, Zhang Ze, Zhang Lifu, Zhao Taolan, Dai Ji, Yang Jialiang, Lan Liang, Luo Ming, Liu Zhaofeng, An Tao, Zhang Bin, He Xiao, Cong Shan, Liu Xiaohong, Zhang Wei, Lewis James P, Tiedje James M, Wang Qi, An Zhulin, Wang Fei, Zhang Libo, Huang Tao, Lu Chuan, Cai Zhipeng, Wang Fang, Zhang Jiabao (2021). Artificial intelligence: a powerful paradigm for scientific research. Innovation (Camb).

[23] Sarker Iqbal H (2021). Machine learning: algorithms, real-world applications and research directions. SN Comput Sci.

[24] Mehta Nishita, Pandit Anil, Shukla Sharvari (2019). Transforming healthcare with big data analytics and artificial intelligence: a systematic mapping study. J Biomed Inform.

[25] Yin Jiamin, Ngiam Kee Yuan, Teo Hock Hai (2021). Role of artificial intelligence applications in real-life clinical practice: systematic review. J Med Internet Res.

[26] Iyamu I, Gómez-Ramírez Oralia, Xu AX, Chang H-J, Watt S, Mckee Geoff, Gilbert Mark (2022). Challenges in the development of digital public health interventions and mapped solutions: findings from a scoping review. Digit Health.

[27] Whitelaw S, Pellegrini D, Mamas M, Cowie M, Van Spall Harriette G C (2021). Barriers and facilitators of the uptake of digital health technology in cardiovascular care: a systematic scoping review. Eur Heart J Digit Health.

[28] Jacob K S (2009). Public health in low- and middle-income countries and the clash of cultures. J Epidemiol Community Health.

[29] Arksey H, O'Malley L (2005). Scoping studies: towards a methodological framework. International Journal of Social Research Methodology.

[30] Yew Sheng Qian, Trivedi Daksha, Adanan Nurul Iman Hafizah, Chew Boon How (2024). Facilitators and barriers of digital health technologies implementation in hospital settings in lower-income and middle-income countries since the COVID-19 pandemic: a scoping review protocol. BMJ Open.

[31] Tricco Andrea C, Lillie Erin, Zarin Wasifa, O'Brien Kelly K, Colquhoun Heather, Levac Danielle, Moher David, Peters Micah D J, Horsley Tanya, Weeks Laura, Hempel Susanne, Akl Elie A, Chang Christine, McGowan Jessie, Stewart Lesley, Hartling Lisa, Aldcroft Adrian, Wilson Michael G, Garritty Chantelle, Lewin Simon, Godfrey Christina M, Macdonald Marilyn T, Langlois Etienne V, Soares-Weiser Karla, Moriarty Jo, Clifford Tammy, Tunçalp Özge, Straus Sharon E (2018). PRISMA Extension for Scoping Reviews (PRISMA-ScR): checklist and explanation. Ann Intern Med.

[32] Jbi (2024). JBI Manual for Evidence Synthesis. JBI Global Wiki.

[33] Feldstein Adrianne C, Glasgow Russell E (2008). A Practical, Robust Implementation and Sustainability Model (PRISM) for integrating research findings into practice. Jt Comm J Qual Patient Saf.

[34] Naeem M, Ozuem W, Howell K, Ranfagni S (2023). A step-by-step process of thematic analysis to develop a conceptual model in qualitative research. International Journal of Qualitative Methods.

[35] (2020). PRISMA Flow Diagram. PRISMA Statement.

[36] Abdulai M, Aldheleai H, Bokhari M (2021). Health care professionals' use of health information systems (HIS) in Indian hospitals. IJCA.

[37] Alboraie M, Abdalgaber M, Youssef N, Moaz I, Abdeen N, Abosheaishaa H, Shokry Mina Tharwat, El-Raey Fathiya, Asfour Sabry Shaaban, Abdeldayem Waleed A, Hassan Adel Ahmed, Mahran Essam Eldeen M O, Tag-Adeen Mohammed, Elshaarawy Omar, Radwan Mohamed Ibrahim, Altonbary Ahmed, Fouad Yasser (2022). Healthcare providers' perspective about the use of telemedicine in Egypt: a national survey. Int J Telemed Appl.

[38] Jiang Yuyu, Sun Pingping, Chen Zhongyi, Guo Jianlan, Wang Shanshan, Liu Fenglan, Li Jinping (2022). Patients' and healthcare providers' perceptions and experiences of telehealth use and online health information use in chronic disease management for older patients with chronic obstructive pulmonary disease: a qualitative study. BMC Geriatr.

[39] Ning Y, Dong Z, Jia Z, Zhao W, Ding Y, Wang Q, Zhu Ruifang, Han Shifan (2023). Development of mobile health-based interventions to promote physical activity in patients with head and neck cancer: a qualitative study. Front Public Health.

[40] Shardha H, Kumar G, Sagar N, Kumar R, Qazi M, Munir S, Tariq Waleed, Maheshwari Payal, Kumar Bhavesh, Tahir Muhammad J, Shrateh Oadi N, Ahmed Ali (2024). Perceptions of telemedicine among healthcare professionals in rural tertiary care hospitals of rural Sindh, Pakistan: a qualitative study. Ann Med Surg (Lond).

[41] Yu-Tong T, Yan Z, Zhen L, Bing X, Qing-Yun C (2022). Telehealth readiness and its influencing factors among Chinese clinical nurses: a cross-sectional study. Nurse Educ Pract.

[42] Mekuria S, Adem HA, Ayele BH, Musa I, Enyew DB (2023). Routine health information system utilization and associated factors among health professionals in public health facilities in Dire Dawa, eastern Ethiopia: a cross-sectional study. Digit Health.

[43] Ngugi Philomena N, Were Martin C, Babic Ankica (2021). Users' perception on factors contributing to electronic medical records systems use: a focus group discussion study in healthcare facilities setting in Kenya. BMC Med Inform Decis Mak.

[44] Tesfa GA, Kalayou MH, Zemene W (2021). Electronic health-information resource utilization and its associated factors among health professionals in Amhara regional state teaching hospitals, Ethiopia. Adv Med Educ Pract.

[45] Tilahun Binyam, Gashu Kassahun D, Mekonnen Zeleke A, Endehabtu Berhanu F, Asressie Moges, Minyihun Amare, Mamuye Adane, Atnafu Asmamaw, Ayele Wondimu, Gutema Keneni, Abera Admas, Abera Mulumebet, Gebretsadik Teklit, Abate Biruk, Mohammed Mesoud, Animut Netsanet, Belay Hiwot, Alemu Hibret, Denboba Wubishet, Gebeyehu Abebaw, Wondirad Naod, Tadesse Lia (2021). Strengthening the national health information system through a capacity-building and mentorship partnership (CBMP) programme: a health system and university partnership initiative in Ethiopia. Health Res Policy Syst.

[46] Baradwan S, Al-Hanawi M Perceived Knowledge, Attitudes, and Barriers Toward the Adoption of Telemedicine Services in the Kingdom of Saudi Arabia: Cross-Sectional Study. JMIR formative research. 2023.

[47] Mussi CC, Luz R, Damázio Dioni da Rosa, Santos EMD, Sun V, Porto Beatriz Silvana da Silveira, Parma Gabriel Oscar Cremona, Cordioli Luiz Alberto, Birch Robert Samuel, Andrade Guerra José Baltazar Salgueirinho Osório de (2023). The large-scale implementation of a health information system in Brazilian university hospitals: process and outcomes. Int J Environ Res Public Health.

[48] Borges do Nascimento Israel Júnior, Abdulazeem H, Vasanthan LT, Martinez EZ, Zucoloto ML, Østengaard Lasse, Azzopardi-Muscat Natasha, Zapata Tomas, Novillo-Ortiz David (2023). Barriers and facilitators to utilizing digital health technologies by healthcare professionals. NPJ Digit Med.

[49] Berardi Chiara, Antonini Marcello, Jordan Zephanie, Wechtler Heidi, Paolucci Francesco, Hinwood Madeleine (2024). Barriers and facilitators to the implementation of digital technologies in mental health systems: a qualitative systematic review to inform a policy framework. BMC Health Serv Res.

[50] Mumtaz H, Riaz MH, Wajid H, Saqib M, Zeeshan MH, Khan SE, Chauhan Yesha Rajendrabhai, Sohail Hassan, Vohra Laiba Iman (2023). Current challenges and potential solutions to the use of digital health technologies in evidence generation: a narrative review. Front Digit Health.

[51] Chidambaram Swathikan, Jain Bhav, Jain Urvish, Mwavu Rogers, Baru Rama, Thomas Beena, Greaves Felix, Jayakumar Shruti, Jain Pankaj, Rojo Marina, Battaglino Marina Ridao, Meara John G, Sounderajah Viknesh, Celi Leo Anthony, Darzi Ara (2024). An introduction to digital determinants of health. PLOS Digit Health.

[52] Sambo L, Simões J, do Rosario O, Martins M, Firth JD, Conlon CP, Cox TM, Firth J, Conlon C, Cox T (2020). Financing Health Care In Low-Income Developing Countries: A Challenge For Equity In Health.

[53] Sureshkumar K, Bindu M, John S, Kamarul Imran M Editorialvidence on low-cost technologies for neurological rehabilitation in low and middle-income countries. Frontiers in Neurology. 2023.

[54] Monaghesh E, Hajizadeh A (2020). The role of telehealth during COVID-19 outbreak: a systematic review based on current evidence. BMC Public Health.

[55] Huang C, Wang J, Wang S, Zhang Y Internet of medical things: A systematic review. Neurocomputing. 2023.

[56] National Institute for Health and Care Excellence (NICE) (2019). Evidence standards framework for digital health technologies. NICE.

[57] (2021). Functional classification, according to their intended use, of digital solutions used in the context of medical and paramedical care. Haute Autorité de Santé.

[58] World Health Organization (WHO) (2018). Classification of digital health interventions v1. WHO.

[59] Food and Drug Administration (FDA) (2024). Digital Health Center of Excellence. FDA.

