# Supplementary material for: Facilitators and Barriers to the Implementation of Digital Health Technologies in Hospital Settings in Lower- and Middle-Income Countries Since the Onset of the COVID-19 Pandemic: Scoping Review
Source: J Med Internet Res. 2025 Mar 6;27:e63482. doi: 10.2196/63482 (PMC11926458; doi:10.2196/63482)
Supplement: Multimedia Appendix 1 [file jmir_v27i1e63482_app1.docx]

**Search Strategies**

**1) List of Search Terms:**

| **Search Terms for Hospital Settings** |
| --- |
| Hospital OR secondary center OR tertiary center OR in-patient OR ward OR hospitalisation |

| **Search Terms for Lower- and Middle-Income Countries (LMIC)** |
| --- |
| Developing countr* OR Emerging econom* OR Third world countr* OR Poverty OR Poor OR Low-income countr* OR Middle-income countr* |

| **Search Terms for Digital Health Technologies** |
| --- |
| Digital health technologies OR digital health interventions OR health information technologies OR health information exchange OR electronic medical record OR electronic health record OR health database OR mobile medical applications OR mHealth OR mobile health OR wireless medical device OR wearable technology OR smart watch OR telemedicine OR telehealth OR teleconsultation OR artificial intelligence OR machine learning OR neural network OR deep learning OR natural language processing OR fuzzy logic OR expert systems OR robotics OR chatbots OR virtual nursing assistants OR risk score OR cyber security OR medical device data systems OR medical device interoperability OR virtual reality OR augmented reality OR 3D printing OR big data analytics OR data mining OR metaverse OR internet of things OR blockchain technology OR drones |

| **Search Terms for Facilitators** |
| --- |
| Facilitators OR enablers OR supporting factor |

| **Search Terms for Barriers** |
| --- |
| Barriers OR obstacles OR challenges OR difficulties OR issues OR preventing factor OR inhibitor |

| **Search Terms for Implementation** |
| --- |
| Implementation OR assimilation OR uptake OR adaptation OR utilisation OR transformation OR usage |

**2) Search Strategy for PubMed Database:**

(Hospital OR “secondary center” OR “tertiary center” OR in-patient OR ward OR hospitalisation) AND (“Developing countr*” OR “Emerging econom*” OR “Third world countr*” OR Poverty OR Poor OR “Low-income countr*” OR “Middle-income countr*”) AND (“Digital health technologies” OR “digital health interventions” OR “health information technologies” OR “health information exchange” OR “electronic medical record” OR “electronic health record” OR “health database” OR “mobile medical applications” OR mHealth OR “mobile health” OR “wireless medical device” OR “wearable technology” OR “smart watch” OR telemedicine OR telehealth OR teleconsultation OR “artificial intelligence” OR “machine learning” OR “neural network” OR “deep learning” OR “natural language processing” OR “fuzzy logic” OR “expert systems” OR robotics OR chatbots OR “virtual nursing assistants” OR “risk score” OR “cyber security” OR “medical device data systems” OR “medical device interoperability” OR “virtual reality” OR “augmented reality” OR “3D printing” OR “big data analytics” OR “data mining” OR metaverse OR “internet of things” OR “blockchain technology” OR drones) AND (Facilitators OR enablers OR “supporting factor”) AND (Barriers OR obstacles OR challenges OR difficulties OR issues OR “preventing factor” OR inhibitor) AND (Implementation OR assimilation OR uptake OR adaptation OR utilisation OR transformation OR usage)

Search Filter: Year: 2020-2023, Language: English

**3) Search Strategy for Scopus Database:**

TITLE-ABS-KEY ((Hospital OR "secondary center" OR "tertiary center" OR in-patient OR ward OR hospitalisation) AND ("Developing countr*" OR "Emerging econom*" OR "Third world countr*" OR Poverty OR Poor OR "Low-income countr*" OR "Middle-income countr*") AND ("Digital health technologies" OR "digital health interventions" OR "health information technologies" OR "health information exchange" OR "electronic medical record" OR "electronic health record" OR "health database" OR "mobile medical applications" OR mHealth OR "mobile health" OR "wireless medical device" OR "wearable technology" OR "smart watch" OR telemedicine OR telehealth OR teleconsultation OR "artificial intelligence" OR "machine learning" OR "neural network" OR "deep learning" OR "natural language processing" OR "fuzzy logic" OR "expert systems" OR robotics OR chatbots OR "virtual nursing assistants" OR "risk score" OR "cyber security" OR "medical device data systems" OR "medical device interoperability" OR "virtual reality" OR "augmented reality" OR "3D printing" OR "big data analytics" OR "data mining" OR metaverse OR "internet of things" OR "blockchain technology" OR drones) AND (Facilitators OR enablers OR "supporting factor") AND (Barriers OR obstacles OR challenges OR difficulties OR issues OR "preventing factor" OR inhibitor) AND (Implementation OR assimilation OR uptake OR adaptation OR utilisation OR transformation OR usage))

Search Filter: Year: 2020-2023, Language: English

**4) Search Strategy for Web of Science Database:**

TS=(Hospital OR "secondary center" OR "tertiary center" OR in-patient OR ward OR hospitalisation) AND TS=("Developing countr*" OR "Emerging econom*" OR "Third world countr*" OR Poverty OR Poor OR "Low-income countr*" OR "Middle-income countr*") AND TS=("Digital health technologies" OR "digital health interventions" OR "health information technologies" OR "health information exchange" OR "electronic medical record" OR "electronic health record" OR "health database" OR "mobile medical applications" OR mHealth OR "mobile health" OR "wireless medical device" OR "wearable technology" OR "smart watch" OR telemedicine OR telehealth OR teleconsultation OR "artificial intelligence" OR "machine learning" OR "neural network" OR "deep learning" OR "natural language processing" OR "fuzzy logic" OR "expert systems" OR robotics OR chatbots OR "virtual nursing assistants" OR "risk score" OR "cyber security" OR "medical device data systems" OR "medical device interoperability" OR "virtual reality" OR "augmented reality" OR "3D printing" OR "big data analytics" OR "data mining" OR metaverse OR "internet of things" OR "blockchain technology" OR drones) AND TS=(Facilitators OR enablers OR "supporting factor") AND TS=(Barriers OR obstacles OR challenges OR difficulties OR issues OR "preventing factor" OR inhibitor) AND TS=(Implementation OR assimilation OR uptake OR adaptation OR utilisation OR transformation OR usage)

Search Filter: Year: 2020-2023, Language: English

**5) Search Strategy for Grey Literature (Google Scholar):**

((Hospital OR “secondary center” OR “tertiary center” OR in-patient OR ward OR hospitalisation) AND (“Developing countr*” OR “Emerging econom*” OR “Third world countr*” OR Poverty OR Poor OR “Low-income countr*” OR “Middle-income countr*”) AND (“Digital health technologies” OR “digital health interventions” OR “health information technologies” OR “health information exchange” OR “electronic medical record” OR “electronic health record” OR “health database” OR “mobile medical applications” OR mHealth OR “mobile health” OR “wireless medical device” OR “wearable technology” OR “smart watch” OR telemedicine OR telehealth OR teleconsultation OR “artificial intelligence” OR “machine learning” OR “neural network” OR “deep learning” OR “natural language processing” OR “fuzzy logic” OR “expert systems” OR robotics OR chatbots OR “virtual nursing assistants” OR “risk score” OR “cyber security” OR “medical device data systems” OR “medical device interoperability” OR “virtual reality” OR “augmented reality” OR “3D printing” OR “big data analytics” OR “data mining” OR metaverse OR “internet of things” OR “blockchain technology” OR drones) AND (Facilitators OR enablers OR “supporting factor”) AND (Barriers OR obstacles OR challenges OR difficulties OR issues OR “preventing factor” OR inhibitor) AND (Implementation OR assimilation OR uptake OR adaptation OR utilisation OR transformation OR usage))

Filter: Year: 2020-2023, screen through the first 200 results
